# Using a two-sample mendelian randomization analysis to explore the relationship between physical activity and Alzheimer’s disease

**DOI:** 10.1038/s41598-022-17207-x

**Published:** 2022-07-28

**Authors:** Bowen Zhang, Xiaowen Huang, Xiliang Wang, Xiaorui Chen, Caifang Zheng, Weihao Shao, Gaili Wang, Weidong Zhang

**Affiliations:** Department of Epidemiology, School of Public Health, Zhengzhou University, Zhangzhou, 450001 Henan People’s Republic of China

**Keywords:** Genetics, Risk factors

## Abstract

Evidence from previous epidemiological studies on the effect of physical activity on the risk of Alzheimer’s disease (AD) is conflicting. We performed a two-sample Mendelian randomization analysis to verify whether physical activity is causally associated with AD. This study used two-sample Mendelian randomization (MR) analysis to estimate the association between physical activity (including overall activity, sedentary behavior, walking, and moderate-intensity activity) and AD. Genetic instruments for physical activity were obtained from published genome-wide association studies (GWAS) including 91,105 individuals from UK Biobank. Summary-level GWAS data were extracted from the International Genomics of Alzheimer’s Project IGAP (21,982 patients with AD and 41,944 controls). Inverse Variance Weighted (IVW) was used to estimate the effect of physical activity on AD. Sensitivity analyses including weighted median, MR-Egger, MR-PRESSO, and leave-one-out analysis were used to estimate pleiotropy and heterogeneity. Mendelian randomization evidences suggested a protective relationship between walking and AD (odds ratio (OR) = 0.30, 95% confidence interval (CI), 0.13–0.68, *P* = 0.0039). Genetically predicted overall activity, sedentary behavior, and moderate-intensity activity were not associated with AD. In summary, this study provided evidence that genetically predicted walking might associate with a reduced risk of AD. Further research into the causal association between physical activity and AD could help to explore the real relationship and provide more measures to reduce AD risk.

## Introduction

Alzheimer’s disease (AD) is the most prevalent cause of dementia^1^. AD is a kind of brain disease that happens to elder people and is responsible for the slow and gradual deterioration in thinking, memory, and language competence, and could also change the personality^1^. AD is the most prevalent neurodegenerative disorder in the world^2^.

There are 44 million individuals living with AD around the world according to the current estimate ^3^. The prevalence of AD in the European population is estimated at 5.05%^4^. The incidence of African Americans is approximately twice that among European Americans^5^. The United States, especially in the West and Southeast might experience the largest growth of AD cases and the number of AD patients could grow to 13.8 million by 2060 years^6^. The growing number of AD cases might cause a huge social-economic burden. Prevention of AD targeting risk factors modification has the potential to curb the increasing number of people living with AD and the increasing economic burden.

Observational studies had identified thirty-three percent of AD cases around the world might be due to several potentially modifiable risk factors after accounting for nonindependence between several risk factors, and the incidence might be decreased by using effective methods targeting to reduce the prevalence of modifiable risk factors such as physical inactivity^7^.

Compared to sedentary lifestyle, long-term exercise could delay the onset of physiological memory loss in middle-aged trained men^8^. Randomized clinical trials (RCT) also had been conducted to investigate whether interventions targeting inactivity could reduce the risk of AD, dementia, or cognitive function. A large and long-term RCT proved that physical activity could improve or at least maintain cognitive functioning in at-risk elder individuals^9^. An intervention study showed that physical activity could provide a modest improvement in cognition for individuals with subjective memory impairment as well^10^. Furthermore, previous meta-analyses including several RCTs also indicated that physical activity intervention showed a statistically significant improvement in cognition of patients diagnosed with AD or slow down the decline of cognition, and different amounts of physical activity can make different effects^11,12^. Additionally, physical activity might be associated with lowering the risk of AD based on a comprehensive meta-analysis^13^.

Though, other long-term RCTs had suggested that physical activity had no significant effects on cognitive decline or might not decrease the prevalence of all-cause dementia^14,15^. No difference in AD risk between physically active and inactive participants was observed in a meta-analysis including 19 prospective observational cohort studies^16^.

Understanding the relative contributions of physical activity to AD is crucial for designing suitable public health interventions to reduce the incidence of AD and decrease the socioeconomic burden. Using observational studies to determine the association might be challenging due to bias of measurement error, confounding, and reverse causation. Therefore, it remains to be elucidated whether the association between physical activity and AD is causal.

Mendelian randomization (MR) analysis using single nucleotide polymorphisms (SNPs) as instrumental variables could be conducted to explore whether the association between physical activity and AD is causal^17^. The study design is similar to RCT since genes are transferred from parents to offspring randomly^17^. All analyses reported in the present study are two-sample MR analyses since SNP-physical activity and SNP-AD associations were extracted from different rather than overlapping samples^18^. The main advantage of using summary-level data from genome-wide association studies (GWAS) in two-sample MR is the increased statistical power, particularly with testing effects on binary disease outcomes^19^.

In this study, we conducted the two-sample MR analysis using summary-level GWAS data on physical activity and AD to make a thorough inquiry about whether physical activity is causally linked with the risk of AD.

## Method

### Study design

We conducted a two-sample MR analysis to explore the association between physical activity and AD. Genetic variants associated with physical activity were selected as genetic instruments for each exposure. The design of our study had three contents: (1) the acquisition of summary-level GWAS data for physical activity and AD; (2) the identification of genetic variants to serve as instrumental variables (IVs); and (3) the estimates of the effects of physical activity on AD.

### Genetic associations with physical activity

Over 100,000 participants (mean age 57.52 years) from UK Biobank, a population-based prospective study^20,21^, who had provided valid email addresses were approached with a wear wrist-worn accelerometer, and 103,712 datasets were received with a median wear-time of 6.9 days (Supplementary Table 1)^22^. After quality control, a total of 91,105 participants of European descent remained for subsequent genome-wide association analysis and summary-level GWAS data used in the present analysis had been adjusted for BMI and sex as covariates^23^. A machine-learning model was used to predict four activity conditions including, sleep, sedentary behavior, walking, and moderate-intensity activity. For instance, sedentary behavior was defined as the energy expenditure score of Metabolic Equivalent of Task (MET) being less than 1.5^24^. More information on genotyping and imputation is shown in Supplementary Method 1. Exposures of interest in our MR analysis were overall activity, walking, sedentary behavior, and moderate-intensity activity. We selected SNPs associated with overall activity, sedentary behavior, and walking at the genome-wide significance level (*p* < 5 $\times$ 10^–8^). Only one SNP (rs568974867) was related to moderate-intensity activity at a genome-wide significance level (*p* < 5 $\times$ 10^–8^). However, rs568974867 was not present in summary-level GWAS data of AD. Therefore, we set the threshold at a suggestive significance level (*p* < 5 $\times$ 10^–6^) for moderate-intensity activity. We eliminated IVs which were associated with other genetic variants (r^2^ threshold < 0.1 and kb = 5,000).

### Genetic associations with Alzheimer’s disease

Genetic associations with AD were obtained from the meta-analyses of GWAS on individuals of European ancestry (n_case_ = 21,982 and n_control_ = 41,944) contributed by the International Genomics of Alzheimer’s Project (IGAP). Details of IGAP were published elsewhere^25^. In brief, in stage 1, IGAP used genotyped and imputed data on 11,480,632 SNPs to meta-analyses GWAS datasets consisting of four consortia: the Alzheimer Disease Genetics Consortium (ADGC); the European Alzheimer's disease Initiative (EADI); the Cohorts for Heart and Aging Research in Genomic Epidemiology Consortium (CHARGE); and the Genetic and Environmental Risk in AD Consortium Genetic and Environmental Risk in AD/Defining Genetic, Polygenic and Environmental Risk for Alzheimer’s Disease Consortium (GERAD/PERADES). Details of the above were shown in Table 1 and Supplementary Method 1. We inferred positive strand alleles by using allele frequencies to harmonize physical activity and AD data. We removed palindromic SNPs with intermediate allele frequency. More details on harmonization were shown in Supplementary Method 2. We did not use proxy SNPs in this research.

**Table 1:** Description of datasets in genome-wide association studies.

Alzheimer's disease	cases			controls		
	N	Percent female	Mean AAO (s.d)	N	Percent female	Mean AAE (s.d)
ADGC	14,428	59.3	71.1 (17.3)	14,562	59.3	76.2 (9.9)
CHARGE	2137	67.3	82.6 (12)	13,474	55.8	76.7 (8.2)
EADI	2240	65	75.4 (9.1)	6631	60.6	78.9 (7.0)
GERAD	3177	64	73.0 (0.2)	7277	51.8	51.0 (0.1)

AAO, age at onset; AAE, age at examination; AGDC, the Alzheimer Disease Genetics Consortium; CHARGE, the Cohorts for Heart and Aging Research in Genomic Epidemiology Consortium; EADI, the European Alzheimer's disease Initiative; GERAD, the Cohorts for Heart and Aging Research in Genomic Epidemiology Consortium.

### Statistical power calculation

Power calculations were conducted using an online web tool for the binary outcome available at https://sb452.shinyapps.io/power/)^26^. The statistical power for our MR relied on several parameters, including type I error of 1.25% after multiple testing corrections, the proportion of variance (R^2^) in the exposure explained by genetic instruments, the “true” causal effect of the physical activity on AD, and the ratio of cases to controls (1 to 1.908). More information is shown in Supplementary Method 3.

### Statistical analysis

In the primary analysis, the fixed-effect inverse variance weighted (IVW) method was used to calculate the overall effects in the absence of heterogeneity^27^. However, if there is heterogeneity between the causal estimates of genetic variances, a random-effects model IVW would be conducted. Cochran’s Q statistic would be computed during MR analysis to estimate whether heterogeneity exists or not. The amount of heterogeneity also was estimated by *I*^2^ statistic^28^.

### Sensitivity analysis

MR was based on three key assumptions^29^. Sensitivity analyses were performed to examine the robustness of the MR results against any potential violation of the three key assumptions. Firstly, genetic variants must be associated with the exposure of interest. The strength of each instrument was measured using the *F* statistics calculated by the formula: F = *R*^2^(N − K − 1)/K(1 − *R*^2^), where *R*^2^ represented the variance in exposures explained by the genetic variance, K represented the number of instruments, and N meant the sample size of the GWAS for the association of the SNP-activity factors^30^. The instrumental variables should be strongly associated with exposures and *F* statistics of all the genetic variants should be larger than the empirical threshold of 10^31^. Secondly, genetic variants must not be associated with potential confounders of the association between the exposure and the outcome, which meant no horizontal pleiotropy. MR-Egger regression, which provided a statistical test for directional pleiotropy according to Egger intercept, was used to assess the potential presence of horizontal pleiotropy^32^. Though, if the Instrument Strength Independent of Direct Effect (InSIDE) assumption was violated, this estimate might increase type I error rates. Therefore, the weighted median estimator was used for sensitivity analysis. This estimate was consistent if up to half of the genetic variants (or variants comprising 50% of the weight for a weighted analysis) were valid IVs^33^. Additionally, we also conducted MR pleiotropy residual sum and outlier (MR-PRESSO) analysis to explore the robustness of our results^34^. MR-PRESSO was a method to detect and correct outliers in IVW linear regression, which might reduce the heterogeneity in the estimates of causal effect by removing outliers. If the MR-PRESSO analysis indicated significant outliers exist, we would remove the outlier variants (with a *P* value less than the threshold in the MR-PRESSO outlier test) and conduct the MR analysis again. At the end of the analysis, a leave-one-out analysis was computed to test the robustness of MR estimates and whether anyone single variant was driving the causal association between exposure and outcome^35^. Thirdly, genetic variants should be associated with AD only through their effects on exposure, not through other pathways. The “negative control” population might be useful to evaluate the exclusion assumption^36^. Sensitivity analyses were not performed for walking as there were only 2 genetic variants.

Results were presented as log odds ratio for comparing with the rest (MET $\le$ 1). The milli-gravity (mg) was used as a unit to report accelerometer-measured physical activity levels. An MR effect was considered significant at a Bonferroni-corrected P-value of 0.05/4 = 0.0125. A *P*-value < 0.05 but above the adjusted *P*-value was considered a suggestive association.

Strengthening the Reporting of Observational Study in Epidemiology (STROBE) MR checklist is provided in Supplementary Information^37^. All analyses were performed with Two-Sample MR package 0.5.5 and MR-PRESSO package 1.0 in R version 4.0.3 (R Foundation for Statistic Computing, Vienna, Austria) (http://www.r-project.org/)^34,38,39^.

### Ethical issues

Additional ethical approval was not required, since this study was based on MR analysis and depended on summary-level GWAS data rather than individual-level data, and the databases used in this analysis had been published or shared before.

## Results

In total, we used 33 SNPs in this multi-instrument MR analysis. Detailed information on genetic variants used in this analysis was shown in Supplementary Table 2. *F* statistics for instruments for overall activity, sedentary behavior, and walking were larger than the empirical threshold of 10, demonstrating the small possibility of weak instrumental variable bias (Table 2). *F* statistics for the instrument for moderate-intensity physical activity was only 2.19, thus suggesting a weak instrument. The statistical power was only 45.3% for sedentary behavior, and the statistical power was between 80% to 1 for another three exposures, including overall activity, walking, and moderate-intensity activity (Table 2).

**Table 2:** Results of F statistic and statistical power for the Mendelian randomization analysis of genetic instruments and the risk of AD.

Exposures	N	K	*R*^2^ (%)	*F* stasistics	Power^a^ (%)
Overall activity	91,105	3	0.06	18.23	83.4
Sedentary behavior	91,105	3	0.06	18.23	45.3
Walking	91,105	2	0.06	27.35	100.0
Moderate-intensity activity (*P* < 5e^−6^)	91,105	25	0.06	2.19	97.7

N, sample size; K, number of genetic instruments; *R*^2^, the proportion of variance in the exposure explained by each genetic instrument.

^a^Statistical power was calculated based on a sample size of 63,926 participants, type I error 1.25% and a ratio of case to control (1 to 1.908).

For overall activity, the random-effect IVW analysis was conducted later due to the heterogeneity (Q_*P*-val_ = 0.024). There was no significant association between genetically predicted overall activity and AD according to the result of IVW analysis (odds ratio (OR) = 0.62; 95% confidence interval (CI), 0.17–2.32, *P* = 0.4805). However, the results computed by the weighted median method proved a suggestive association (OR = 0.35; 95% CI 0.13–0.92, *P* = 0.0362). There was an absence of directional pleiotropy according to MR-Egger regression (intercept = 0.19, 95% CI 0.05–0.33, *P* = 0.234). Besides, the MR estimate of ME-Egger was very different from others (OR = 0.00; 95% CI 0.00–0.14, *P* = 0.2207). The results of the null relationship were consistent before and after removing the outlier using MR-PRESSO (Table 3). Plots of leave-one-out analysis (Supplementary Fig. 1) demonstrated that there was potentially influential SNP driving the link between overall activity and AD. Therefore, we need to carefully interpret the result and draw a cautious conclusion. A Scatter plot was shown in Supplementary Fig. 2.

**Table 3:** MR Estimates for the effects of physical activity on AD risk in IGAP datasets.

Exposure	Outcome	Outcome units	Method	SNPs	*P*	OR	95%CI	Q	Q_df	Q p-val	I2	MR Egger			MR-PRESSO			
												Intercept	95%CI	Intercept pval	Global test *P*	SNPs	OR	*P*
Overall activity	Alzhemier's disease	Log odds ratio	MR Egger	3	0.2207	0.00	(0.00,0.14)	0.67	1	0.412	0.00	0.19	(0.05,0.33)	0.234	0.004	2	1.02 (0.16, 6.58)	0.996
			IVW	3	0.4805	0.62	(0.17,2.32)	7.43	2	0.024	0.73							
			IVW (mre)	3	0.4805	0.62	(0.17,2.32)											
			Weighted median	3	0.0362	0.35	(0.13,0.92)											
Sedentary behavior	Alzheimer’s disease	Log odds ratio	MR Egger	3	0.8147	1.40	(0.06,41.95)	0.01	1	0.922	0.00	− 0.01	(− 0.10,0.09)	0.916	0.185			
			IVW	3	0.4131	1.33	(0.68,2.60)	0.00	2	0.987	0.00							
			IVW (mre)	3	0.0000	1.33	(1.23,1.43)											
			Weighted median	3	0.5103	1.30	(0.60,2.80)											
Walking	Alzheimer’s disease	Log odds ratio	IVW	2	0.0039	0.30	(0.13,0.68)	0.68	1	0.411	0.00	NA	NA	NA	NA			
			IVW (mre)	2	0.0004	0.30	(0.15,0.59)											
Moderate-intensity activity	Alzheimer’s disease	Log odds ratio	MR Egger	25	0.8918	1.06	(0.49,2.28)	26.06	23	0.298	0.12	− 0.01	(− 0.03,0.01)	0.450	0.165			
			IVW	25	0.1371	0.80	(0.59,1.07)											
			IVW (mre)	25	0.1371	0.80	(0.59,2.07)	26.73	24	0.317	0.10							
			Weighted median	25	0.9210	0.98	(0.65,1.46)											

SNPs, single nucleotide polymorphisms; CI, confidence interval; OR, odds ratio; Q, Cochran Q statistics; IVW, inverse variance weighted; WM, weighted median; MR-Egger, Mendelian randomization-Egger; MR-PRESSO,MR pleiotropy residual sum and outlier.

*P*_het_: *P*-values of Cochran’s Q test for heterogeneity were shown.

*P*_int_: *P*-values of MR-Egger regression test on the intercept.

**Figure 1:**
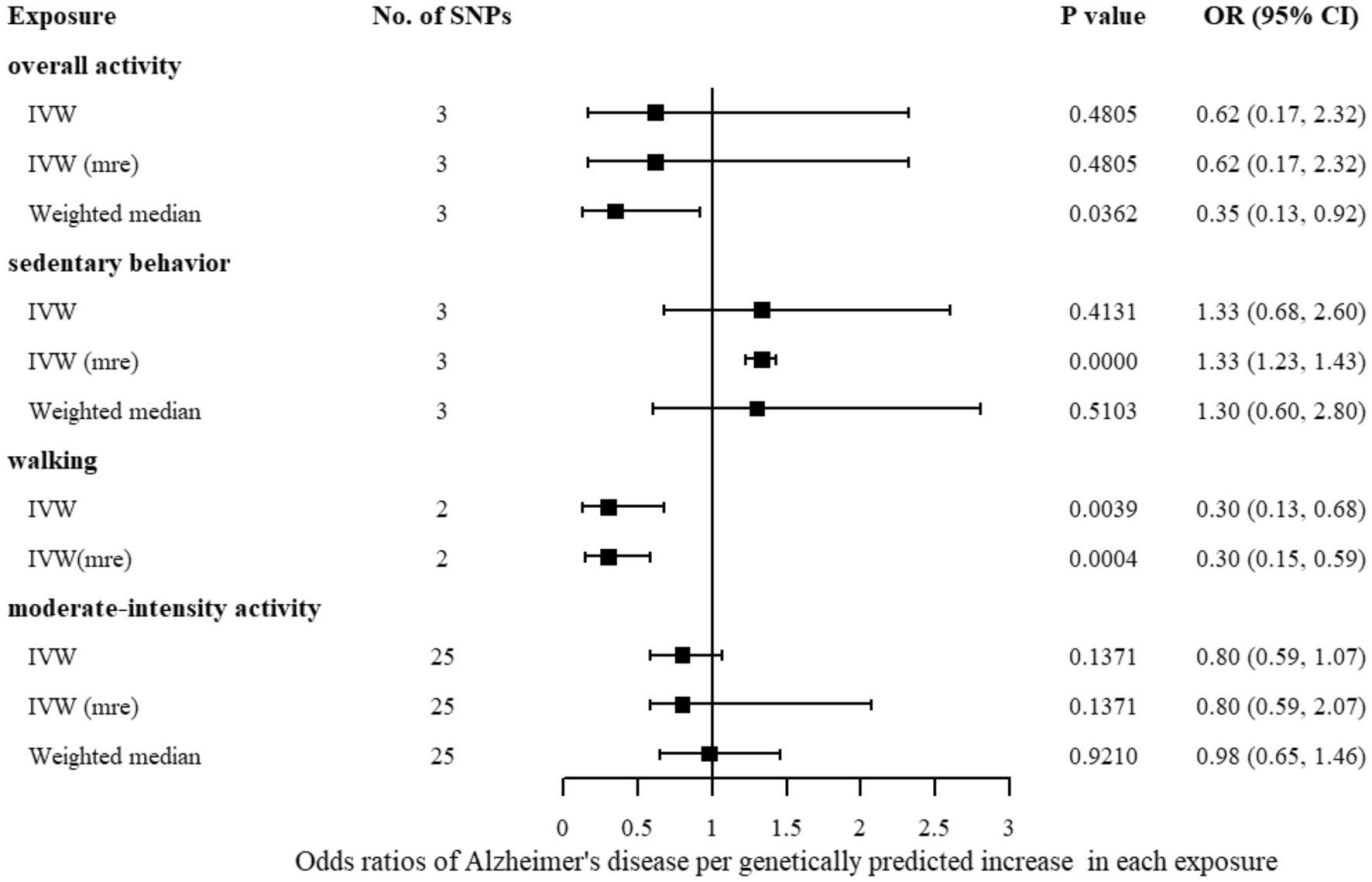
Forest plot for MR analysis of the causal effect of physical activity on Alzheimer’s disease. SNPs, single nucleotide polymorphisms; No. of SNPs, number of SNP; OR, odds Ratio; 95% CI, 95% confidence interval; IVW, inverse variance weighted.

The result of random-effect IVW proved sedentary behavior might be associated with a higher risk of AD (OR = 1.33; 95% CI 1.23–1.43, *P* = 2.0188 $\times$ 10^–12^) (Table 3, Fig. 1). Though fixed-effect IVW and weighted median analysis did not show evidence of a causal link. The MR-Egger intercept and MR-PRESSO did not show evidence of pleiotropy with sedentary behavior. Plots of leave-one-out analysis demonstrated no potentially influential SNPs driving the link between sedentary behavior and AD (Supplementary Fig. 1). A Scatter plot of SNP effects on exposure versus their effects on AD was shown in Supplementary Fig. 2.

The IVW regression showed that an increase in walking time might decrease the risk of AD (OR = 0.30, 95% CI 0.13–0.68, *P* = 0.0039). The result was consistent with the random-effect IVW method (OR = 0.30; 95% CI 0.15–0.59, *P* = 0.0004). There was still a significant association after multiple testing corrections. The results were shown in Fig. 1. There was no heterogeneity according to Cochran’s Q test (*P* = 0.411) (Table 3). However, no other sensitivity analyses were conducted due to the limited number of IVs.

As for moderate-intensity activity, using 25 genetic instruments, linear MR analysis demonstrated no causal effects of moderate-intensity activity on AD risk (OR = 0.80; 95% CI 0.59–1.07, *P* = 0.1371), which was consistent with results in the weighted median method. Our analysis suggested no significant evidence of horizontal pleiotropy (as indicated by MR-Egger regression intercept close to zero and *P* = 0.450, Supplementary Fig. 2). The result of MR-PRESSO analysis indicated that no outliers existed (Table 3). The result of the leave-one-out analysis (Supplementary Fig. 1) showed potentially influential SNP driving the link between moderate-intensity activity. Results were shown in Fig. 1.

## Discussion

In this two-sample MR analysis, we examined the effects of physical activity, including overall activity, sedentary behavior, walking, and moderate-intensity activity, on the risk of AD. Genetically predicted overall activity, sedentary behavior, and moderate-intensity activity were not associated with AD. Higher levels of walking might be beneficial for preventing AD.

For overall activity, the results of the IVW analysis (both fixed-effect and random-effect) suggested the absence of an association between exposure and AD. Nevertheless, the weighted median demonstrated a suggestive relationship. Besides, there was a huge difference between the effect sizes from IVW and ME-Egger analyses. While the MR-Egger estimate might be imprecise and influenced by outlying genetic variants, MR-PRESSO analysis did suggest outlier SNPs existed. There was still a null association between overall activity and AD when we excluded the potential outlier. Based on the different results drawn by sensitivity analyses, we should be cautious to conclude that overall activity does not influence the risk of AD.

There was no heterogeneity exiting for sedentary behavior according to Cochran’s Q statistic. We mainly focused on the MR estimate of the fixed-effect IVW method, which revealed a positive relationship between sedentary behavior and AD. Higher levels of sedentary behavior seemed related to a higher risk of AD, which indicated a deleterious effect of sedentary behavior on AD. However, consistent with the results of sensitivity analyses, the association was not significant. Sedentary behavior was defined as an activity that had a MET energy expenditure score of $\le$ 1.5 and usually occurs at sitting, lying, and reclining status^24^. Nonetheless, other behaviors such as driving (MET < 2.5) and some instances of non-desk work were categorized as sedentary behavior in the original GWAS analysis which might be the explanation for the absence of association in our MR analysis^23^.

We found that the risk of AD decreases by proximately 30% for each 1-SD increase in genetically predicted walking levels. Although we only used 2 SNPs as instrumental variables for walking, these 2 SNPs explained a 0.12% variance in walking and they fulfilled the criterion of not being weak instrumental variables (*F* statistics > 10)^31^. Besides, the statistical power of the MR analysis for walking was sufficient. Therefore, low statistical power and weak instruments are unlikely to explain our results. However, sensitivity analysis could not be conducted to verify the independence and exclusion restriction assumption which might influence our results due to the limited number of SNPs.

The results of MR analysis did not demonstrate a relationship between moderate-intensity activity and AD. The null association might be influenced by a bias of weak instruments (*F* statistic = 2.19) due to violations of instrumental assumption. Secondly, even when using large samples (n = 91,105), results using weak instruments might be biased towards the null in the two-sample MR analysis^36^. That was also a well-powered MR result since moderate-intensity activity showed significant power (> 80%).

Our results were consistent with previous studies which suggested that physical activity was associated with a decreased AD risk^13,40–42^. The result from the previous analysis indicated exercise was related to a 28% decreased risk of AD was similar to our results^13^. The meta-analysis, which included 3345 participants had point estimates that suggested a protective effect of physical activity. The result of this meta-analysis finding an OR for the development of AD was 0.60 (95% CI 0.51–0.71) compared to inactive individuals^40^. Our study confirmed the finding of a clinical trial that suggested physical activity was associated with a reduced risk of AD (OR = 0.48; 95% CI 0.17–0.85)^43^.

The mechanism might be explained as follows: both animal and human research had shown that exercise resulted in beneficial changes to brain structure and function accompanying cognitive function^44,45^. Physical activity might modulate amyloid β (Aβ) turnover, inflammation, the synthesis and release of neurotrophins, and cerebral blood flow (CBF)^46,47^. Besides, activity can influence cognition through different pathophysiological processes such as the immune system, endothelial function, cerebrovascular insufficiency, oxidative stress, and neurotoxicity^48^.

It was worth mentioning that our results indicated walking rather than moderate-intensity activity is related to the reduced AD risk. The possible explanation might be the small sample size and the young age of subjects from previous research^49^. For AD, symptoms usually occurred after age 60^1^. Secondly, the beneficial effect of an exercise intervention on cognitive function was driven by the types of activity^50^. Previous research might mix the effect of moderate-intensity activity with light and vigorous activity making it difficult to isolate the effects of moderate-intensity activity^51,52^. Further, moderate-intensity activity was defined by energy expenditure score in our MR analysis. The classification criteria for moderate-intensity activity in the previous research were inconsistent with the present MR analysis^45^. Apart from that, physical activity intervention in other research included not only aerobic exercise but also strength, and balance exercise^42,53,54^. The beneficial effect of moderate-intensity activity on cognitive function might pair with other elements. Our conclusion was supported by previous studies, which indicated that walking might enhance cognitive performance and suggested walking as little as 1.5 h weekly was beneficial for cognition in healthy elderly^55^. Furthermore, walking was not only positively correlated with emotional health, and memory, but also was beneficial for reducing depression scores, anxiety, and lower psychological stress^56–59^. Walking could even stabilize the progressive cognitive dysfunctions in AD patients^60,61^. Thus, we suggested that walking might be an effective strategy for AD prevention.

The results contrasted the finding of other studies that reported no difference in AD risk between physically active and inactive participants. The reasons for the difference between our MR analysis result and other research might be explained as follows. Firstly, previous studies could not exclude the potential effects caused by some biases, including unmeasurable confounders, reverse causality, and recall biases. Besides, for different randomized controlled trials, the intervention of physical activity or aerobic exercise and follow-up periods might be quite different. Moreover, there might be heterogeneity among these studies, especially in the different methods of measurement used for cognitive function. Secondly, most previous studies focused on the outcome of the cognitive improvement in people with AD or mild cognitive impairment, not the risk of incidence^62,63^. Thirdly, there were limited subjects in previous RCT studies^64,65^. Therefore, it would be hard to compare the effects of physical activity on AD risk.

Our results were contradictory to a previous MR study which suggested that physical activity did not affect the risk of development of AD^66^. The possible reason might be as follows: previous MR studies used moderate-to-vigorous physical activity (MET $\ge$ 6) as exposure, which might be different from the effects of daily activity. Physical activities were divided into more detailed categories, including sedentary behavior, walking, and moderate-intensity activity in the present MR analysis.

This study had several strengths. Firstly, daily physical activities including sedentary behavior and walking were recorded by activity trackers and were more precise than self-report data. Genetic variants identified in GWAS analysis based on self-reported data were at least partly influenced by reporting bias^67,68^. Secondly, based on the design of the present two-sample MR analysis, there might be less bias from confounding which would have been the case if both physical activity and AD GWAS were performed in the same sample. Thirdly, the influence of confounding factors on the studied association was reduced due to the strict selection of IVs. Fourthly, the previous MR analysis used average accelerations and the fraction of accelerations > 425 milligravities as exposure measures, the latter meaning vigorous physical activity (METs $\ge$ 6) only^66^. More physical activity statuses were analyzed in our study. Besides, various methods, including IVW, MR-Egger, weighted-median, and MR-PRESSO were performed in this MR analysis to robust our conclusion. Finally, population stratification could be decreased in the present study as the AD dataset was restricted to participants of European ancestry.

Our study had certain limitations. Firstly, the GWAS of exposure consisted of participants aged 55–74 years with higher socioeconomic and physical health status, which might affect the results^23^. Besides, our results assumed that the sample used to define the IVs for physical activity and the samples from the IGAP consortium used to estimate the genetic association with AD are both from European ancestry. Hence, our results might not be suited to all populations. Thirdly, we cannot exclude the possibility of the sample overlap since both physical activity and AD used individuals from Europe. Therefore, that might generate bias in our analysis. A further limitation is that the number of genetic variants used for walking was limited for sensitivity analysis. Another limitation is that the studies included in IGAP used different diagnostic criteria for AD, but all cases met standard criteria for possible, probable, or definite AD. Hence certain misclassification might be inevitable. Sixthly, these exposure datasets recorded by wrist-worn accelerometer co-occur and interact across daily life. Multivariable MR analysis could be conducted to explore the relationship between physical activity and AD to make the results more precise^69^. Seventhly, the association between moderate-intensity activity and AD might be biased by weak instruments (*F* statistic = 2.19). Besides, the final MR assumption (the exclusion assumption) had not been evaluated. We did not search through the PhenoScanner to screen genetic variants which might be related to confounding factors^70^. We just conducted MR-PRESSO analysis to detect and remove outliers. We additionally calculated statistical power for our MR analysis. We had sufficient power to detect the effects of exposures on AD, except for sedentary behavior. Therefore, we should draw our conclusion with caution. Finally, the two-sample MR analysis assumed a linear relationship between physical activity and AD. In this research, we could not investigate the nonlinear effects of physical activity. Nevertheless, there could be a nonlinear relationship, such as U- or J- shaped association, between physical activity and AD. Therefore, further research could be needed to detect the causal factors of AD.

In summary, this study provided evidence that genetically predicted walking might associate with a reduced risk of AD. The elderly could use walking as the main way of commuting in daily life to reduce the risk of AD. Further research into the causal association between risk factors and AD could help to explore the real relationship and provide more measures to reduce AD risk. Mechanisms between physical activity and AD might need more investigation. More high-quality RCTs with fewer methodological issues and heterogeneity also are needed to back up our present findings.

## Supplementary Information

Supplementary Information 1.

Supplementary Tables.

## Data Availability

The datasets analyzed during the current study can be downloaded from [10.5287/bodleian:yJp6zZmdj] and GWAS Catalog [http://ftp.ebi.ac.uk/pub/databases/gwas/summary_statistics/GCST007001-GCST008000/GCST007511/].
