# Supplementary material for: Using a two-sample mendelian randomization analysis to explore the relationship between physical activity and Alzheimer’s disease
Source: Sci Rep. 2022 Jul 28;12:12976. doi: 10.1038/s41598-022-17207-x (PMC9334579; doi:10.1038/s41598-022-17207-x)
Supplement: Supplementary file 1 — Supplementary Information 1. [file 41598_2022_17207_MOESM1_ESM.pdf]

Using a Two-sample Mendelian Randomization Analysis to Explore the Relationship between Physical Activity and Alzheimer's Disease

Bowen Zhang [1], Xiaowen Huang [2], Xiliang Wang [3], Xiaorui Chen [4], Caifang Zheng [5], Gaili Wang [6], Weihao Shao [7], Weidong Zhang [8 *]

1 Bowen Zhang, Department of Epidemiology, School of Public Health, srxiugao300hengzhou University, Zhangzhou 450001, Henan, People's Republic of China.

2 Xiaowen Huang, Department of Epidemiology, School of Public Health, Zhengzhou University, Zhangzhou 450001, Henan, People's Republic of China.

3 Xiliang Wang, Department of Epidemiology, School of Public Health, Zhengzhou University, Zhangzhou 450001, Henan, People's Republic of China.

4 Xiaorui Chen, Department of Epidemiology, School of Public Health, Zhengzhou University, Zhangzhou 450001, Henan, People's Republic of China.

5 Caifang Zheng, Department of Epidemiology, School of Public Health, Zhengzhou University, Zhangzhou 450001, Henan, People's Republic of China.

6 Gaili Wang, Department of Epidemiology, School of Public Health, Zhengzhou University, Zhangzhou 450001, Henan, People's Republic of China.

7 Weihao Shao, Department of Epidemiology, School of Public Health, Zhengzhou University, Zhangzhou 450001, Henan, People's Republic of China.

8 Weidong Zhang, Department of Epidemiology, School of Public Health, Zhengzhou University, Zhangzhou 450001, Henan, People's Republic of China.

* Corresponding to: Weidong Zhang, email: imooni@163.com.

Supplement Information Content

Supplementary Method 1. Details of summary-level GWAS data of physical activity and Alzheimer's disease.

Supplementary Method 2. Details of harmonize progress.

Supplementary Method 3. MR Power Calculation.

Supplementary Figure 1. Plots of "leave-one-out" analyses for MR analyses of the causal effect of physical activity on Alzheimer's disease.

Supplementary Figure 2. Scatter plots for MR analyses of the causal effect of physical activity on Alzheimer's disease, with the slope of each line corresponding to the estimated MR effect per method.

Reference.

Supplementary Method 1. Details of Summary-level GWAS data of physical activity and Alzheimer's disease.

Summary-level GWAS data of physical activity

A total of 103,702 participants from the UK Biobank agreed to wear an Axivity AX3 wrist-worn accelerometer for 7 days between 2013 and 2015(*1*). After quality control, a total of 91,105 participants of European descent remained for subsequent genome-wide association analysis and summary-level GWAS data used in the present analysis had been adjusted for BMI and sex as covariates(*2*). Machine-learning model, using a balanced random forest with Markov confusion matrices, was conducted to identify activity states, which was achieved with 79% accuracy (kappa = 0.68). Device non-wear-time was categorized as consecutive stationary episodes lasting for at least 60 min. Those non-wear segments of data were imputed with the average of similar time-of-day data points, for each behavior prediction, from different days of the measurement(*2*).

There were two similar genotyping arrays for the UK Biobank, including Applied Biosystems UK BiLEVE Axiom Array and A UK Biobank Axiom Array. The marker content of the UK Biobank Axiom array was chosen to capture genome-wide genetic variation and short insertions and deletion (INDELs). Genotyping was carried out by Affymetrix Research Services Laboratory. Markers that failed quality control were removed. Phasing on the autosomes was performed by using SHAPEIT3. The UK Biobank used UK10K haplotype, 1000 Genomes Phase 3, and Haplotype Reference Consortium (HRC) reference panels as a reference set for imputation. The 1000 Genomes Phase 3 was used predominantly to help with the phasing of samples with non-European ancestry. The Haplotype Reference Consortium (HRC) reference panel was used as the main reference panel since it consisted of an assailable set of European haplotypes. Imputed data using the merged UK10K and1000 Genomes Phase 3 reference panels was combined with that from the HRC panel. Further, HRC imputation was used when the SNP existed in both panels. Imputation was carried out with the IMPUTAE4 program(*3*). SNPs with MAF $< 0.1\%$ (< 83 M) and imputation $R^2 < 0.3$ (~ 9 k) were excluded for quality control. BOLT-LMM was used to perform linear mixed

model analysis. Candidate variants that deviated from Hardy-Weinberg equilibrium ($p < 1\times 10^{-7}$) were excluded through PLNK (version 1.9). Input SNPs were mapped by position to genes acquired from protein-coding genes obtained through Ensembl 2018(*4*).

Summary-level GWAS data of Alzheimer's disease

IGAP used genotyped and imputed data on 11,480,632 SNPs to meta-analyses GWAS datasets consisting of four consortia: the Alzheimer Disease Genetics Consortium (ADGC); the European Alzheimer's disease Initiative (EADI); the Cohorts for Heart and Aging Research in Genomic Epidemiology Consortium (CHARGE); and the Genetic and Environmental Risk in AD Consortium Genetic and Environmental Risk in AD/Defining Genetic, Polygenic and Environmental Risk for Alzheimer's Disease Consortium (GERAD/PERADES).

Standard quality control was performed on all data sets individually. Individuals with non-European ancestry according to principal components analysis of ancestry-information markers were excluded(*5*). Each dataset was phased and imputed to the 1,000 Genomes Project. All reference population haplotypes were used for the imputation, which could improve the accuracy of imputation for low-frequency variants. Single variants-based association analysis employed an additive genotype model adjusted for age (defined as age-at-onset-for cases and age-at-last exam for controls), sex, and population substructure.

Supplementary Method 2.

Harmonization progress

To perform MR, the effect of SNPs on AD and physical activity must be harmonized to be relative to the same allele. Therefore, we harmonized the alleles and effects between physical activity and AD. During the harmonization progress, we tried to infer positive strand alleles, using allele frequencies for palindromes. Then we removed SNPs for being palindromic with intermediate allele frequency(*6, 7*).

Supplementary Method 3.

MR Power Calculation

We estimated statistical power for our MR analysis by an online web tool for the binary outcome (https://sb452.shinyapps.io/power/)(*8*). Statistical power for MR given a specific sample size based on several parameters, including the proportion of variants (R^2) in the exposure explained by genetic instruments; the causal effect of the exposure on the outcome, and the ratio of cases to controls (for binary outcome).

Using the IGAP GWAS sample size (n = 63,926) and the ratio of cases to controls (1 to 1.908), we calculated statistical power for our MR analysis. We had 83.4% power to detect the relationship between overall activity and AD. For sedentary behavior, there might be not sufficient power (45.3%). For 2 SNPs on walking, we calculated sufficient power (100%) to detect the effects of digital-device walking on AD. For 25 genetic variants on moderate-intensity behavior, we calculated sufficient power (97.7%) to detect the effects of moderate-intensity activity on AD.

Supplementary Figure 1. Plots of “leave-one-out” analyses for MR analyses of the causal effect of physical activity on Alzheimer’s disease. (A) overall activity – AD, (B) sedentary behavior – AD, (C) moderate-intensity activity – AD.

Note: No “leave-one-out” analysis was conducted for MR analysis on causal effects of walking due to the limited number of SNPs.

Supplementary Figure 1-(A) Plots of “leave-one-out” analyses for MR analyses of the causal effect of overall activity on Alzheimer’s disease.

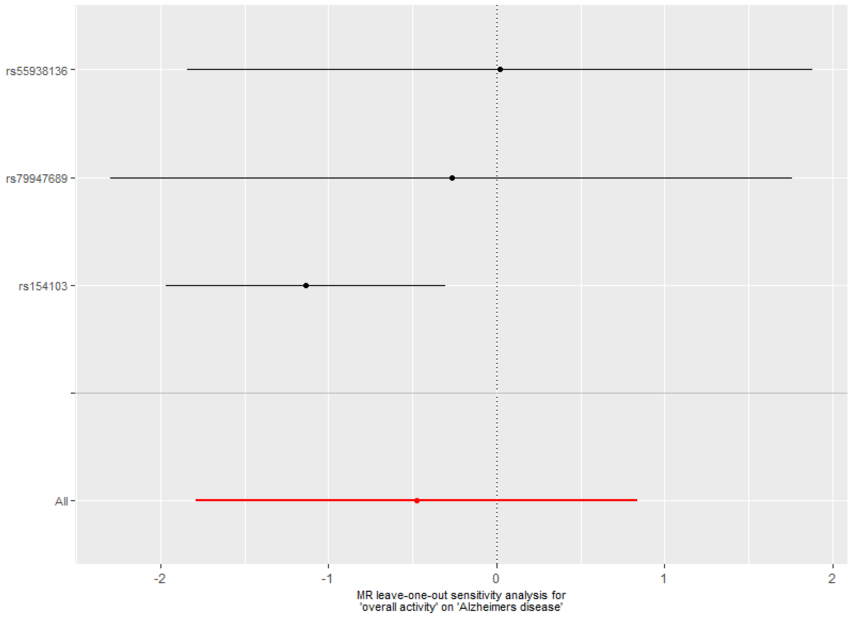

Supplementary Figure 1- (B) Plots of “leave-one-out” analyses for MR analyses of the causal effect of sedentary behavior on Alzheimer’s disease.

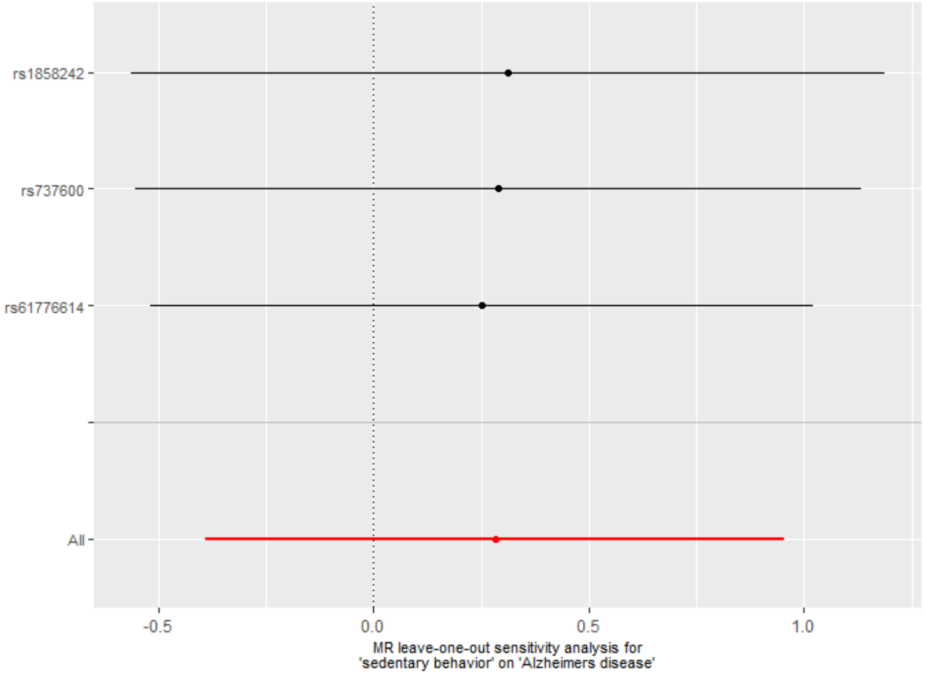

Supplementary Figure 1- (C) Plots of “leave-one-out” analyses for MR analyses of the causal effect of moderate-intensity activity on Alzheimer’s disease.

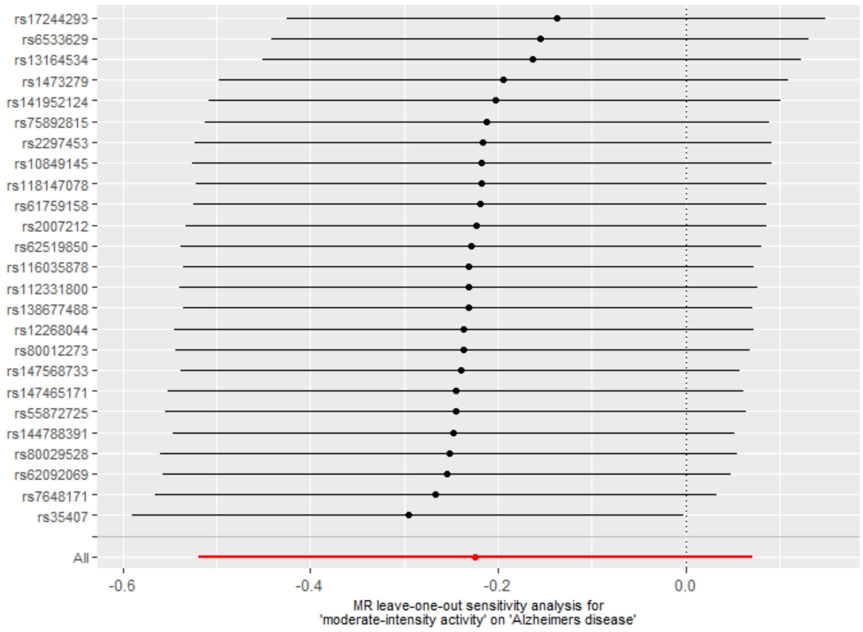

Supplementary Figure 2. Scatter plots for MR analyses of the causal effect of physical activity on Alzheimer's disease, with the slope of each line corresponding to the estimated MR effect per method. (A) overall activity – AD, (B) sedentary behavior – AD, (C) moderate-intensity activity – AD.

Note: No scatter plot was conducted for MR analysis on causal effects of walking due to the limited number of SNPs.

Supplementary Figure 2-(A) Scatter plots for MR analyses of the causal effect of overall activity on Alzheimer's disease, with the slope of each line corresponding to the estimated MR effect per method.

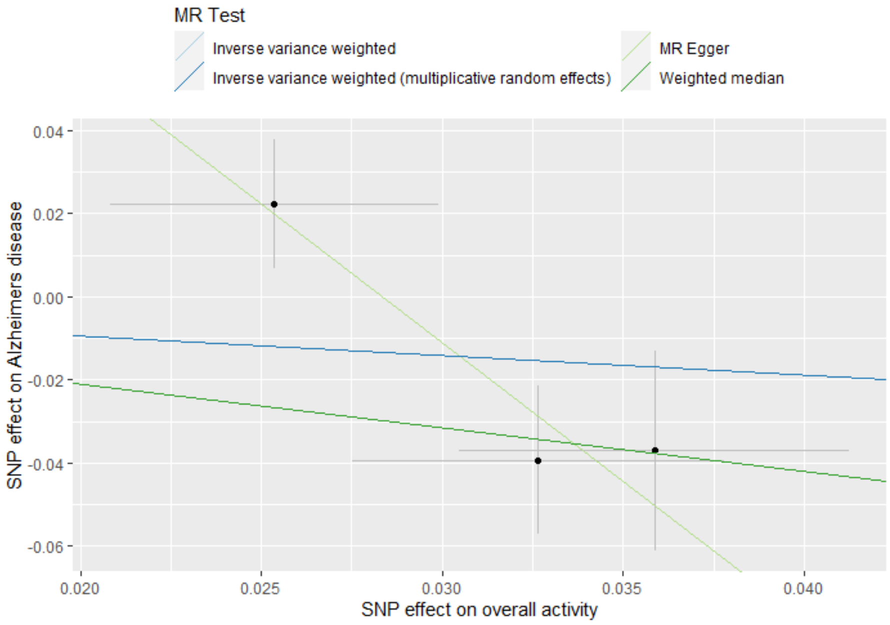

Supplementary Figure 2-(B) Scatter plots for MR analyses of the causal effect of sedentary behavior on Alzheimer's disease, with the slope of each line corresponding to the estimated MR effect per method.

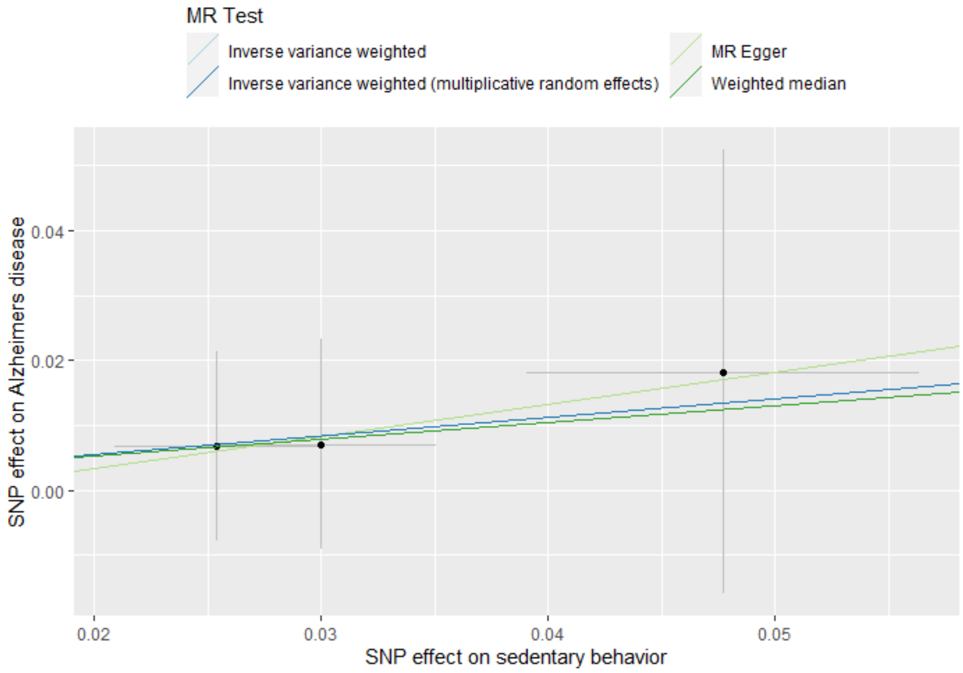

Supplementary Figure 2-(C) Scatter plots for MR analyses of the causal effect of moderate-intensity activity on Alzheimer's disease, with the slope of each line corresponding to the estimated MR effect per method.

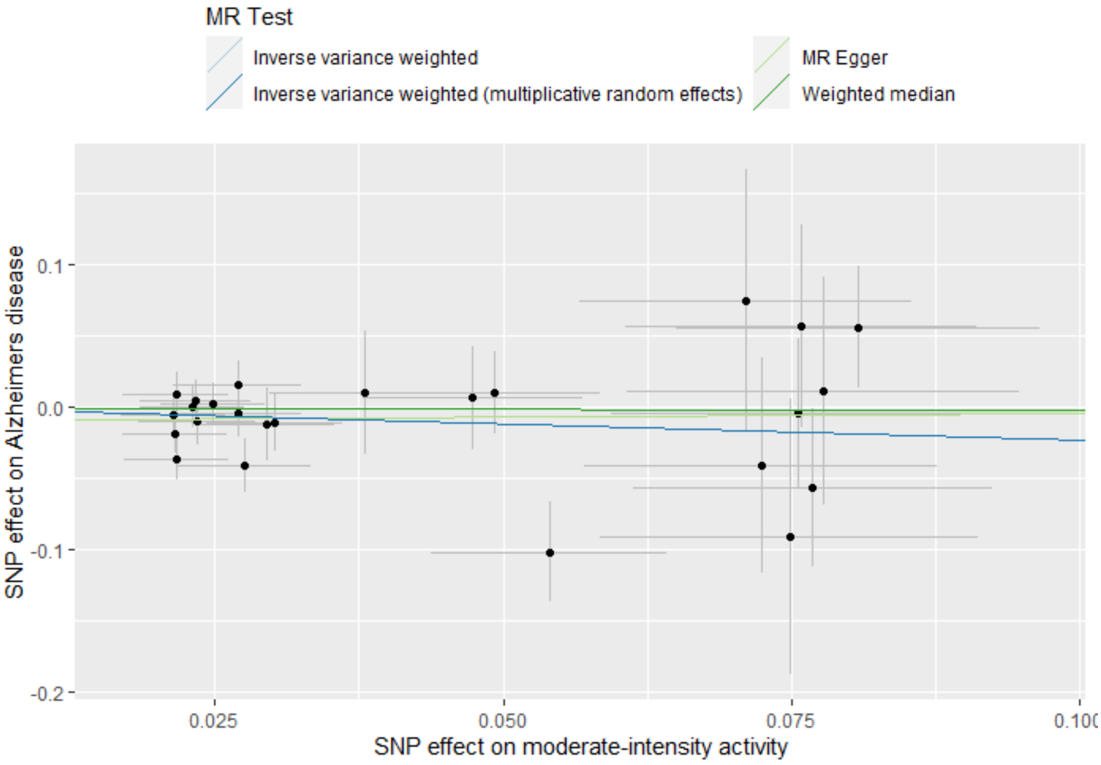

Reference

1. A. Doherty *et al.*, Large Scale Population Assessment of Physical Activity Using Wrist Worn Accelerometers: The UK Biobank Study. *PLoS One* **12**, e0169649 (2017).
2. A. Doherty *et al.*, GWAS identifies 14 loci for device-measured physical activity and sleep duration. *Nat Commun* **9**, 5257 (2018).
3. C. Bycroft *et al.*, The UK Biobank resource with deep phenotyping and genomic data. *Nature* **562**, 203-209 (2018).
4. D. R. Zerbino *et al.*, Ensembl 2018. *Nucleic Acids Res.* **46**, D754-d761 (2018).
5. B. W. Kunkle *et al.*, Genetic meta-analysis of diagnosed Alzheimer's disease identifies new risk loci and implicates Abeta, tau, immunity and lipid processing. *Nat. Genet.* **51**, 414-430 (2019).
6. G. Hemani *et al.*, The MR-Base platform supports systematic causal inference across the human phenome. *Elife* **7**, (2018).
7. G. Hemani, K. Tilling, G. Davey Smith, Orienting the causal relationship between imprecisely measured traits using GWAS summary data. *PLoS Genet.* **13**, e1007081 (2017).
8. S. Burgess, Sample size and power calculations in Mendelian randomization with a single instrumental variable and a binary outcome. *Int. J. Epidemiol.* **43**, 922-929 (2014).
